# How I treat rhabdomyolysis-induced AKI? A different perspective

**DOI:** 10.1177/03913988241269508

**Published:** 2024-09-02

**Authors:** Gabriella Bottari, Isabella Guzzo

**Affiliations:** 1 Pediatric Intensive Care Unit, Bambino Gesù Children’s Hospital, IRCCS, Rome, Italy; 2 Division of Nephrology, Bambino Gesù Children’s Hospital, IRCCS, Rome, Italy

Dear Editor,

An increasing number of studies suggest the effectiveness of hemoadsorption (HA) in managing severe rhabdomyolysis.^1,2^ An effective reduction in myoglobin levels through HA has been reported, compared to standard hemodialysis with high-flux membranes and high cutoff membranes.^1,2^ Myoglobin is the main target of extracorporeal blood purification therapies due to its molecular weight of 17 kDa, which allows it to be effectively removed by HA. Creatinine Kinase (CK), composed 43 kDa dimers, is a biochemical biomarker for rhabdomyolysis.^3^ CK and myoglobin seem to be correlated,^1^ but the most studies^1,3^ identify myoglobin as a better biomarker and some authors suggest a limited removal of CK even with HA.^2^

**Figure 1.**
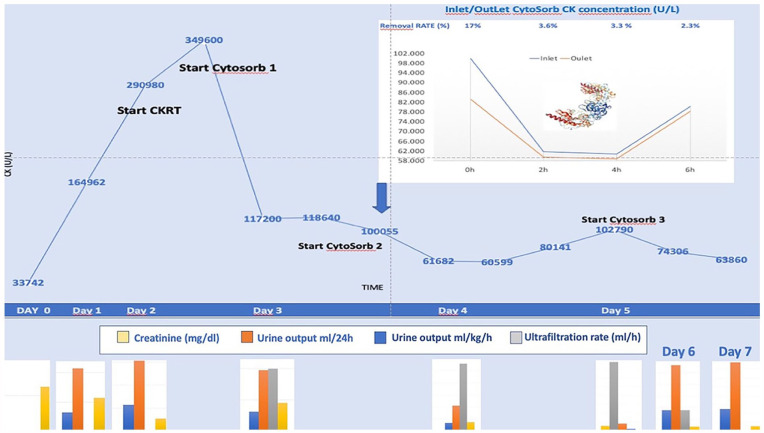
Upper section of the figure shows time course of Creatinine Kinase (CK) during the extra-corporeal therapy with Continuous Kidney Replacement Therapy (CKRT) and Cytosorb hemoadsorption. The white window depicts the time course of CK measured at the inlet and the outlet of the cartridge between Time 0 (0 h) and after 6 h (6 h). At the different time points is reported the CK removal ratio of the cartridge (calculated as [Concentration at baseline (CB0) − Concentration at the end of the treatment (Cend)/CB0 × 100]). Bottom section of the figure shows the histograms ad different time points for creatinine blood level (yellow bars), urine output measured in ml in 24 h (orange bars), urine output measured as ml × kg/h (blue bars), ultrafiltration rate of CKRT (ml/h).

In this regard, we report the case of a 3-year-old girl weighing 13 kg admitted to the PICU for altered sensorium, hyponatremia, metabolic acidosis, and hyperkalemia diagnosed as severe viral rhabdomyolysis. The patient, initially treated conservatively with hydration and urine alkalization, demonstrated refractoriness to this therapy. CK values increase from 33,742 to 290,980 U/L within 24 h. Real-time myoglobin measurement was unavailable. Thus, Continuous Kidney Replacement Therapy (CKRT) treatment was initiated using a high-flux (ANST69) continuous veno-venous hemodiafiltration (CVVHDF) mode, using a pre-filter reinfusion and an effluent dose of 2000 ml/h × 1.73 m^2^ with Prismaflex^®^ machine. The CKRT circuit was flushed with saline solution and primed with blood. Anticoagulation was managed with regional citrate anticoagulation with a starting citrate dose of 2.5 mmol/L and with an aim of circuit calcium of 0.3–0.4 mmol/l and patient calcium of 1.1–1.25 mmol/l. We observed a further increase in CK values over the next 4 h, reaching a maximum of 349,600 U/L. Subsequently HA with a Cytosorb column (CytoSorb^®^, CytoSorbents Inc, New Jersey, USA) in combination with CKRT was initiated with a reduction of CK to 117,200 U/L in the next 6 h. The column was then replaced after 12 h following a CK plateau at 118,640 U/L. During treatment with the second column, we sampled CK values before (inlet) and after (outlet) the column, as shown in Figure 1. After 12 h, we further changed the column and CK values reduced to 63,860 U/L. Thus, we decided to continue treatment exclusively with CKRT, observing a progressive reduction in CK values to <10,000 U/L. The patient was discharged from the PICU on the 10th day with no dependence on dialysis.

Our experience suggests that the observed reduction in CK (inlet versus outlet HA) is not only a result of a reduction in endogenous production as reported by previous authors^2^ but also consequence of active removal by the column, as already described for myoglobin. However, during a still active endogenous production cartridge saturation occurred in our patient very early (4 h). Furthermore, in the presence of severe rhabdomyolysis, changing of the columns should be performed at an appropriate time for the kinetics of saturation, primarily based on the entity of endogenous muscle breakdown processes. This clinical case shows as, despite the severe rhabdomyolysis, the patient maintained spontaneous diuresis throughout the extra-corporeal treatment. The pathogenesis of AKI in rhabdomyolysis is multifactorial and related to myoglobin intratubular precipitation but also to inflammatory causes with mediators effectively removed by adsorption columns. Thus, based on both our clinical experience and the available literature data,^4^ early implementation of HA in the presence of severe rhabdomyolysis could potentially prevent the development of AKI.

In conclusion, we advocate for a “paradigm shift” in managing severe rhabdomyolysis, recommending HA for CK levels >12,000 U/L and >10,000 ng/ml myoglobin, with column changes timed based on clinical condition and biomarker levels.
